# Clinicopathological Characteristics, Treatment, and Prognosis of Rarely Primary Epididymal Adenocarcinoma: A Review and Update

**DOI:** 10.1155/2017/4126740

**Published:** 2017-12-20

**Authors:** Zi-jun Zou, Ying-ming Xiao, Zhi-hong Liu, Ruo-chen Zhang, Jia-yu Liang, Yong-quan Tang, Yi-ping Lu

**Affiliations:** ^1^Institute of Urology, Department of Urology, West China Hospital, Sichuan University, Chengdu, Sichuan 610041, China; ^2^Department of Urology, Sichuan Cancer Hospital & Institute, Chengdu, Sichuan 610041, China; ^3^Department of Urology, Fujian Provincial Hospital, Fuzhou, Fujian 350001, China

## Abstract

Primary epididymal adenocarcinoma (PEA) is exceedingly rare. Only 22 cases had been published worldwide by 2008; nearly 80% of these cases were reported before 2007. In order to investigate the current clinical status of PEA, we search for relevant literatures with “epididymis and adenocarcinoma” and “epididymal and adenocarcinoma” as keywords published between January 1997 and November 2017 in PubMed. As a result, 17 cases are identified. We review these cases and summarize new and important perspectives about the clinicopathological characteristics, diagnosis, treatment, and prognosis of PEA in the present review.

## 1. Introduction

Most lesions of the epididymis, including inflammatory and most neoplastic diseases [1], are benign, but rare malignant lesions still should be taken into account in the differential diagnosis. Epididymal cancer is rare, accounting for 0.03% of all male cancers. 51% of malignant tumors of the epididymis are primary or metastatic carcinomas and 44% are sarcomas [2]. Primary epididymal adenocarcinoma (PEA) is one of the epithelial malignancies and even rarer. Ganem and colleagues found only 22 cases were reported worldwide in 2008 [3], but they considered the actual number of PEA patients might be less. Because, in some old cases, PEAs were inadequately described or poorly illustrated, some other tumors such as papillary cystadenoma, adenomatoid tumor and metastatic tumors might be misdiagnosed as PEA [3, 4]. Recently, we searched using “epididymis and adenocarcinoma” and “epididymal and adenocarcinoma” as keywords in PubMed and identified 17 cases about PEA published in the past 20 years between January 1997 and November 2017 [3–16]. Due to its rarity, the natural characteristics of PEA are unclear currently, and diagnosis and treatment have to be based on putative principles. In order to reveal the characteristics and current clinical status of PEA, we summarize the latest 17 cases in terms of clinicopathological characteristics, diagnosis, treatment, and prognosis in the review.

## 2. Clinical Features

PEA patients range in age from 27 to 81 years (mean, 58 years), and nearly 70% of the patients are older than 50 years in the included cases. Ganem et al. previously reported that about 57% of PEA patients were older than 50 years [3]. PEA can occur on either side of the epididymis, but no bilateral PEA is reported. History of the disease ranges from 15 days to 40 years before diagnosis, and half of the patients have it more than 6 months. If an epididymal mass enlarges suddenly and rapidly, it may indicate malignancy [3, 5]. Graham et al. suspected that PEA could arise as a malignant transformation of a benign papillary cystadenoma of the epididymis (PCE) [6]. Relationship between PEA and von Hippel-Lindau disease (VHLD) is not clear with only one case revealing such association [7], and thus detection of the VHL gene mutation is not recommended routinely at diagnosis. TP53 gene mutation was detected in a PEA [8].

80% of the patients complain of scrotal swelling or palpable mass. About 33% of the patients suffer from intrascrotal pain. The pain may be associated with the invasive growth pattern of the disease. 38.5% of PEAs are accompanied by hydrocele.

41.2% of the PEA patients have localized disease, and others have regional lymph node (LN) and/or distant organ metastasis. Anatomically, there are two lymphatic drainage routes of the epididymis; one is from the epididymis caput and corpus to the preaortic nodes and the other is the epididymis cauda to the external iliac nodes [17]. Retroperitoneal and pelvic LN metastasis are both observed in PEA. Six out of ten metastatic PEAs have retroperitoneal LN metastasis [4, 9–12], and one patient has pelvic LN metastasis [4]. Therefore, retroperitoneal and pelvic LN can be defined as regional LN of PEA. Lung, bone, and abdominal organ are common distant metastatic sites; the metastatic probability is 50%, 33.3%, and 33.3% correspondingly. A positron emission tomography and computed tomography scan (PET/CT) for metastatic evaluation is useful [9], but negative findings can not preclude metastasis [10]. Clinical features are summarized in Table 1.

## 3. Pathological Features

Diagnosis of PEA mainly depends on pathological examination. Macroscopically, the reported maximum diameter of mass ranges from 0.4 to 7 cm (mean, 3.3 cm). It is whitish or tan-yellow and hard. Necrosis, or invasion of surrounding soft tissue, testis, or spermatic cord, is likely to be observed [3, 4, 8].

Histological features of PEA are variable and mixed (as shown in Table 2). Tubular, papillary, tubulopapillary, cystopapillary, or solid structure can be observed. Cytoplasm of tumor cells is water-clear, amphophilic, or eosinophilic. These mentioned features can also be observed in a benign PCE. However, typical malignant features, such as mitotic figures, nuclear pleomorphism, necrosis, and/or invasive growth pattern, should be observed in a PEA, while absent in a PCE.

Immunohistochemical analysis (IHC) can assist in diagnosing a PEA (as shown in Tables 3 and 4). Markers specific for epithelial tumors, such as cytokeratin and epithelial membrane antigen (EMA), are positive in PEA [9, 11, 13, 14]. PAX2 is important for the development of the Wolffian ducts and thus it is positive in tumors originating from Wolffian ducts-associated organs, containing PEA [5, 18]. Clear cell papillary cystadenocarcinoma of the epididymis, which has positive CK7, negative RCC marker, and focal immunoreactivity to CD10, can be distinguished from metastatic clear cell renal cell carcinoma (ccRCC) [5]. By contrast, CK7 staining is reportedly negative in mucinous and poorly differentiated adenocarcinoma of the epididymis [8, 9]. Prostate specific antigen (PSA), placental alkaline phosphatase (PAP), S100, vimentin, alpha fetoprotein (AFP), calretinin, and leukocyte common antigen staining can be performed to exclude other types of tumors including metastatic prostate cancer, melanoma, sarcoma, testicular tumor, mesothelioma, and lymphoma. However, it should be noted that IHC markers may be expressed in diverse primary cancers, which are not typically associated with the marker expression. IHC results must be interpreted in the context of the overall morphologic features.

Microscopical and IHC features of clear cell papillary cystadenocarcinoma of the epididymis are similar to those of clear cell papillary renal cell carcinoma (ccpRCC) [5]. Though no cases about metastatic ccpRCC in the epididymis were reported to our knowledge, the metastatic disease still should be noted. In order to distinguish the two diseases, specific markers for PEA need to be further explored.

## 4. Differential Diagnosis 

Prior to the diagnosis of PEA, metastatic adenocarcinomas should be considered, containing those originating from kidney [7], gastrointestine [19, 20], pancreas [21], bile duct [22], and prostate [23], especially in patients having a history of malignant neoplasms. Pindoria et al. reported that a patient, having a history of multiple renal carcinomas and VHLD, suffered from primary papillary cystadenocarcinoma of the epididymis with testicular metastasis [7]. In this case, metastatic RCC in the testis and epididymis were precluded by histological review, IHC, and imaging detection [7]. Prostate cancer is the most common tumor metastasizing to the epididymis with 27 cases reported [24]. Digital rectal examination, serum PSA detection, transrectal ultrasound, and prostate magnetic resonance imaging are helpful to uncover primary prostate cancer. Similarly, other metastatic adenocarcinomas can be excluded by multidisciplinary evaluation including pathological, endoscopic, and radiological examinations.

The majority of epididymal neoplasms are benign, and adenomatoid tumor is the most common among them. In addition, PCE, leiomyoma, and lipoma can be seen. The differentiation between a PEA and these benign tumors is not difficult by microscopically morphologic and IHC analysis.

## 5. Treatment

Standardized treatment for PEA is lacking. Epididymal malignancies account for approximately 25% of all epididymal tumors [3]. If an epididymal tumor is strongly suspected, transinguinal exploration is needed. Radical orchiectomy (RO) should be performed, when intraoperative frozen section indicates malignant tumor, both of the epididymis and testis are abnormal, and/or epididymal mass can not be distinguished from the testis. RO promises en bloc tumor excision and is beneficial for subsequent lymphadenectomy because of lymph drainage of the epididymis going along with the spermatic cord into abdomen. Simple excision of PEA may lead to positive surgical margin and recurrence [3], and transscrotal approach carries the risk of lymphatic violation [6].

Anatomically, retroperitoneal lymph node dissection (RPLND) and pelvic lymph node dissection (PLND) may play an important and even curable role in treatment for PEA. Jones and colleagues reported that a PEA patient, with two positive retroperitoneal LNs (a total of 25 nodes were dissected), did not have a relapse for 30 years after early RPLND [4]. Staník et al. recommended performing RPLND not only in PEA with lymphadenopathy but also as prophylactic treatment in clinical N0 disease [9]. Patients with retroperitoneal LN metastasis seem to be more possible to benefit from primary RPLND, even in the case of obviously clinical retroperitoneal LN metastasis at diagnosis [4, 9, 10], than from secondary surgery in time of retroperitoneal recurrence during the follow-up period [4, 11]. The role of PLND is still unknown because no relevant cases were reported. In the only case reporting pelvic LN metastasis, external beam radiation was performed instead of PLND [4]. Direct anatomic route for lymphatic drainage from the epididymis to the inguinal LN is absent, and thus inguinal lymph node dissection (ILND) seems not to be necessary as a primary treatment for PEA. ILND was reportedly performed in 3 cases [4, 6, 14], of which 2 had no evidence of inguinal metastasis postoperatively [4, 14]. Though inguinal LN metastasis was found in Graham et al.'s case [6], it was probably secondary to the change of lymphatic drainage route due to previously transscrotal spermatocelectomy.

The evidence of radiotherapy (RT) and chemotherapy for PEA is limited. In one case about a locally relapsed adenocarcinoma of the epididymis, complete remission was achieved 3 months after RT and later the effect lasted 42 months [10]. Platinum-based regimens were used as a first-choice chemotherapy for advanced disease in 3 cases [10, 11, 14]; transient positive effect on disease progression was observed [9, 11].

## 6. Prognosis

Prognostic factors of PEA are uncertain. Distant organ metastasis probably indicates poor prognosis. Arisan and colleagues reported a PEA patient, with lateral acetabulum and spleen and liver metastasis, died 6 months after diagnosis [14]. Distant metastasis is also the main cause of death after surgery; patients usually die 6 to 8 months later after distant metastasis [4, 11]. Chemotherapy may delay the tragic end [8].

Subclinical [10] and clinical retroperitoneal LN metastasis at first diagnosis [9] may have better prognosis than retroperitoneal LN metastasis found during the follow-up period after primary RO [4, 11]. No retroperitoneal LN metastasis found after RPLND may indicate good prognosis [15].

TP53 gene mutation is likely to be related to poor prognosis of a PEA [8]. The reported treatment and prognosis of PEA are summarized in Table 5.

## 7. Conclusion

PEA is an exceedingly rare malignant tumor. Its diagnosis and treatment are still challenges. Correct diagnosis depends on comprehensively clinical examinations, pathological analysis, and a close follow-up. Early PEA may be cured by radical orchiectomy and appropriate lymph node dissection. Platinum-based chemotherapy and radiotherapy may be transiently effective on late and relapsed PEA. Distant organ metastasis probably indicates poor prognosis.

## Figures and Tables

**Table 1:** Clinical features of 17 included cases of primary epididymal adenocarcinoma.

Variables	Number (%^#^)
Age	
Range	27–81 yr
Mean	58 yr
≤50 yr	5 (31.3)
>50 yr	11 (68.8)
Unknown case	1
History	
Range	0.5–480 mo
≤6 mo	4 (50)
>6 mo, ≤ 2 yr	2 (25)
>2 yr	2 (25)
Unknown case	9
Clinical presentation	
Swelling or mass	12 (80)
Scrotal pain or discomfort	5 (33.3)
Incidental finding	1 (6.7)
Flank and lower abdominal discomfort	1 (6.7)
Infertility	1 (6.7)
Unknown case	2
Side	
Left	4 (36.4)
Right	7 (63.6)
Unknown case	6
Maximum diameter	
Range	0.4–7 cm
Mean	3.3 cm
Unknown case	3
Hydrocele	
Yes	5 (38.5)
No	8 (61.5)
Unknown case	4
Stage	
No metastasis	7 (41.2)
RLN metastasis	7 (41.2)
Distant metastasis	6 (35.3)
Both of RLN and distant metastasis	3 (17.6)
Distant metastatic site	
Lung	3 (50)
Bone	2 (33.3)
Abdominal organ	2 (33.3)

^#^The proportion is calculated in the cases which can offer relevant data. RLN: regional lymph node (including retroperitoneal and pelvic lymph node).

**Table 2:** Histological characteristics and regional metastatic status of primary epididymal adenocarcinoma were reported in the literatures from 2007 to 2017.

Ref.	Diagnosis	Tissue Structure	Cell morphology	Nucleus	Cytoplasm	Invasion	Tumor stroma	Proven metastasis
Graham et al. 2017 [6]	Adenocarcinoma	Papillary and gland-like	—	Small, punctate, and round nuclei	Pale eosinophilic	—	—	ILN

Pindoria et al. 2016 [7]	Papillary cystadenocarcinoma	Papillary structures projecting into cystic spaces; cystic and solid sheets	Cuboid to columnar, polygonal	—	Pale eosinophilic to clear	Spermatic cord	—	Ipsilateral testis

Urabe et al. 2016 [13]	Adenocarcinoma	Nest-like and tubular pattern; lobulated proliferation	—	—	—	—	Fibrotic and inflammatory	No

Gupta et al. 2015 [8]	Mucinous adenocarcinoma	Cystic spaces; variably sized tubular glands with intraluminal papillae; complex tubulocystic structures with mucin; calcification and necrosis	Frank goblet cell differentiation	Nuclear stratification, moderate nuclear pleomorphism, coarse chromatin, and frequently prominent nucleoli; identified mitosis	Intracytoplasmic mucin	Periepididymal soft tissue, testis, rete testis and spermatic cord	—	No

Nozawa et al. 2014 [5]	Clear cell papillary cystadenocarcinoma	Solid nests and tubular structure; necrosis	—	Small round nuclei (a part); nuclear atypia and occasional mitosis (a part)	Clear to eosinophilic	Testicular capsule and surrounding soft tissues	—	No

Staník et al. 2012 [9]	Poorly differentiated adenocarcinoma	Microacinar and solid, sporadically cribriform; no necrosis	—	Mainly round with finely dispersed chromatin, with sporadic nuclei variability such as hyperchromasia, prominent nucleoli and monstrous nuclei; high mitosis	Large clear vacuoles without evident mucus secretion	Testis	Fibrous septa	RPLN

Soumarová et al. 2012 [10]	Adenocarcinoma	Tubular, papillary, tubulopapillary and cystopapillary structures alternating with solid structures	Cuboid, columnar and epitheloid	High atypical mitosis	Some contain clear cytoplasm	Endolymphatic, endovenous, (peri)endoneural and pseudocapsule tumour permeation	Pseudocapsules and incomplete fibrous septa	RPLN

Arisan et al. 2004 [14]	Small differentiated adenocarcinoma	Irregular adenoid structures; solid spherical or papillary pattern proliferation	Big	Pleomorphic vesicular nucleus, definite nucleolus; some mitosis	—	Seminal cord	—	No

Hayashi et al. 2003 [16]	Moderately differentiated adenocarcinoma	Cord- and nest-like or complex glandular pattern; necrosis	—	Hyperchromatic, pleomorphic; increased mitosis	—	Testis	—	No

Chauhan et al. 2001 [11]	Poorly differentiated adenocarcinoma	—	—	—	A strong PAS staining	Epididymal tubules	—	RPLN

Ganem et al. 1998 [3]	Well differentiated adenocarcinoma	Microglandular	—	—	—	Perineural space	—	No

Jones et al. 1997 [4]	Adenocarcinoma	*NO. 1 + 2, *variably sized, simple tubules or complex tubular glands with intraluminal papillae; necrosis (in one patient) *NO. 3 + 4*,large cysts contain lightly eosinophilic secretions and had complex, arborizing papillary structures projecting into them	*NO. 1 + 2*,cuboidal or rarely columnar *NO. 3 + 4*,columnar	*NO. 1 + 2*,mildly to moderately atypical nucleus, visible nucleoli; infrequent mitosis *NO. 3 + 4*,infrequent mitosis	*NO. 1 + 2*,clear or rarely lightly amphophilic *NO. 3 + 4*,lightly amphophilic, eosinophilic or clear cytoplasm	*NO. 1 + 2*,epididymal muscular wall, periepididymal soft tissue, or both. *NO. 3 + 4*,—	*NO. 1 + 2*,focally desmoplastic stroma *NO. 3 + 4*,—	*NO. 1 + 4*,RPLN *NO. 2 + 3*,No

ILN: inguinal lymph node; RPLN: retroperitoneal lymph node; — means that contents were not mentioned in the literatures.

**Table 3:** Markers of immunohistochemical analysis were used for the diagnosis and differentiation of primary epididymal adenocarcinoma in different cases.

Ref.	Type	Immunohistochemical markers
Graham et al. 2017 [6]	Adenocarcinoma	CK7(+), CD10(+), Mesothelin(+), CAIX(+), PSA(−), PROSAP(−), CK20(−), CDX2(−), WT1(−), SALL4(−), Glypican3(−), CK5(−), Calretinin(−), and S100(−)
Urabe et al. 2016 [13]	Adenocarcinoma	EMA(+), CAM5.2(+), C-KIT(−), PLAP(−), AFP(−), CD30(−), HCG(−), Inhibin(−), Calretinin(−), WT1(−), HBME1(−) and PSA(−)
Gupta et al. 2015 [8]	Mucinous adenocarcinoma	CK7(−), TTF-1(−), CK20(+), Villin(+), CDX2(−), P53(+) and PAS(+)
Nozawa et al. 2014 [5]	Clear cell papillary cystadenocarcinoma	CK7(+), CD10(+), PAX2(+), Vimentin(+), CAIX(+), Vinculin(+), AMACR(−), RCC marker(−), GST-*α*(−) and C-KIT(−)
Staník et al. 2012 [9]	Poorly differentiated adenocarcinoma	CK7(−), TTF-1(−), CD10(+), CK AE1/AE3(+), EMA(+), CA19-9(+), HBME1(+), Inhibin(−), Calretinin(−), PLAP(−), CD30(−), Melan-A(−), CA125(−) and CEA(−)
Arisan et al. 2004 [14]	Small differentiated adenocarcinoma	PSA(−), CEA(+) and EMA(+)
Hayashi et al. 2003 [16]	Moderately differentiated adenocarcinoma	AFP(−), CEA(−) and CA19-9(−)
Chauhan et al. 2001 [11]	Poorly differentiated adenocarcinoma	CK(+), PAS(+), PAP(−), LCA(−), PSA(−), Vimentin(−) and S100(−)
Ganem et al. 1998 [3]	Well differentiated adenocarcinoma	PSA(−), PAP(−), CEA(+), Vimentin(−) and Leu-M1(−)
Jones et al. 1997 [4]	Adenocarcinoma	CK(+), EMA(+), CEA(−), AFP(−), Leu-M1(−), B72.3(−) and Ber-EP4(−)

**Table 4:** Summary of immunohistochemical marker expression status in primary epididymal adenocarcinoma.

Marker	Primary epididymal adenocarcinoma	
	Positive/total cases	Negative/total cases
CEA	2/5	3/5
PSA	0/5	5/5
CK7	2/4	2/4
EMA	4/4	0/4
AFP	0/3	3/3
Calretinin	0/3	3/3
CD10	3/3	0/3
Vimentin	1/3	2/3
CA19-9	1/2	1/2
CAIX	2/2	0/2
CD30	0/2	2/2
CDX2	0/2	2/2
CK	2/2	0/2
CK20	1/2	1/2
C-KIT	0/2	2/2
HBME1	1/2	1/2
Inhibin	0/2	2/2
Leu-M1	0/2	2/2
PAP	0/2	2/2
PAS	2/2	0/2
PLAP	0/2	2/2
S100	0/2	2/2
TTF-1	0/2	2/2
WT1	0/2	2/2
AMACR	0/1	1/1
B72.3	0/1	1/1
Ber-EP4	0/1	1/1
CA125	0/1	1/1
CAM5.2	1/1	0/1
CK5	0/1	1/1
CK AE1/AE3	1/1	0/1
Glypican	0/1	1/1
GST-*α*	0/1	1/1
HCG	0/1	1/1
LCA	0/1	1/1
Melan-A	0/1	1/1
Mesothelin	1/1	0/1
P53	1/1	0/1
PAX2	1/1	0/1
PROSAP	0/1	1/1
RCC	0/1	1/1
SALL4	0/1	1/1
Villin	1/1	0/1
Vinculin	1/1	0/1

**Table 5:** Treatment and prognosis of primary epididymal adenocarcinoma reported in the literatures from 2007 to 2017.

Ref.	Primary treatment	IFP	Interval time^*※*^	Secondary treatment	Follow-up time and prognosis
Graham et al. 2017 [6]	Epididymectomy, RO, scrotectomy, resection of the inguinal mass and ILND	No use	—	—	12 mo; right ILN and suspected pulmonary metastasis

Pindoria et al. 2016 [7]	A biopsy	No use	After having a child by IVF treatment	RO and onco-micro TeSE	—

Urabe et al. 2016 [13]	RO	No use	—	—	10 mo; no evidence of metastasis and recurrence

Gupta et al. 2015 [8]	RO	No use	2 yr	Chemotherapy (capecitabine)	30 mo; bilateral pulmonary metastasis was found 2 yr after surgery and the lesions were stable 6 mo later

Nozawa et al. 2014 [5]	RO	—	—	—	—

Staník et al. 2012 [9]	RO	No use	4 mo	RPLND and chemotherapy (paclitaxel and carboplatin)	20 mo; no evidence of metastasis and recurrence

Soumarová et al. 2012 [10]	Orchiectomy, RPLND	—	6 mo	Palliative RT	48 mo; scrotal recurrence was found 6 mo after surgery and the lesion was complete remission 42 mo after RT

Yang et al. 2010 [15]	RO, RPLND	Yes	—	—	At least 10 mo; no evidence of metastasis and recurrence

Arisan et al. 2004 [14]	RO, unilateral ILND and chemotherapy (cisplatin and etoposide)	No use	—	—	Patient died of right lateral acetabulum and spleen and liver metastasis after 6 mo

Hayashi et al. 2003 [16]	RO	No use	—	—	17 mo; no evidence of metastasis and recurrence.

Chauhan et al. 2001 [11]	RO	Yes	1 yr	RPLND and radiochemotherapy (2nd); palliative chemotherapy (3rd)	Patient died after 30 mo. RLN metastasis occurred 1 yr after initial surgery. Multiple bone metastasis occurred 1 yr after secondary treatment

Ganem et al. 1998 [3]	Transscrotal epididymectomy	No use	1 mo	Radical orchiectomy and hemiscrotectomy	18 mo; no evidence of metastasis and recurrence.

Jones et al. 1997 [4]	①RO ②RO and RT ③RO ④RO, RPLND and ILND	①No use ②No use ③No use ④No use	① 1 yr ② — ③ — ④ —	① RPLND ② — ③ — ④ —	① Patient died after 20 mo. RLN metastasis occurred 1 yr after initial surgery. Bilateral lung metastasis occurred 8 mo later. ② Patient died of extensive abdominal and PLN metastases after 6 mo. ③ — ④ 30 yr; no evidence of metastasis and recurrence

IFP: intraoperative frozen pathology; ILN: inguinal lymph node; ILND: inguinal lymph node dissection; IVF: in vitro fertilization; onco-micro TeSE: microsurgical testicular sperm extraction in cancer patients; PLN: pelvic lymph node; RPLN: retroperitoneal lymph node; RPLND: retroperitoneal lymph node dissection; RO: radical orchiectomy; RT: radiotherapy. ^*※*^interval  time indicates the time between primary and secondary treatment.
